# Neuromodulation Techniques for Headache Management

**DOI:** 10.3390/life14020173

**Published:** 2024-01-24

**Authors:** Noora Reffat, Carolina Pusec, Scott Price, Mayank Gupta, Philippe Mavrocordatos, Alaa Abd-Elsayed

**Affiliations:** 1 School of Medicine and Public Health, University of Wisconsin, Madison, WI 53705, USA; reffat@wisc.edu (N.R.);; 2 Department of Neurology, University of Wisconsin Health, Madison, WI 53705, USA; 3 Kansas Pain Management & Neuroscience Research Center, Kansas City, KS 66214, USA; 4 Swiss Pain Institute, 1003 Lausanne, Switzerland; 5 Department of Anesthesiology, University of Wisconsin Health, Madison, WI 53705, USA

**Keywords:** headache, migraine, neuromodulation, pain management

## Abstract

This narrative review aims to summarize evidence regarding the current utilization and future applications of neuromodulation in patients with headaches, with special attention paid to migraine and chronic cluster headache. A search was conducted in PubMed in August of 2023 to survey the current literature on neuromodulation for the treatment of headache. In total, the search yielded 1989 results, which were further filtered to include only systematic reviews published between 2022 to 2023 to capture the most up-to-date and comprehensive research on this topic. The citation lists of these articles were reviewed to find additional research on neuromodulation and supplement the results presented in this paper with primary literature. Research on the use of neuromodulation for the treatment of headache has predominantly focused on four neuromodulation techniques: peripheral nerve stimulation (PNS), transcranial magnetic stimulation (TMS), deep brain stimulation (DBS), and spinal cord stimulation (SCS). Outcome measures reported in this article include impact on migraine and headache frequency and/or pain intensity, adverse effects of the neuromodulation technique, and associated costs, when available. We found that neuromodulation has developed utility as an alternative treatment for both chronic cluster headaches and migraines, with a reduction in frequency and intensity of headache most elucidated from the articles mentioned in this review.

## 1. Introduction

Headaches compose nearly 3% of all chief complaints in the emergency room, posing significant burden on both the healthcare system and those who suffer from them [1,2]. Unlike many other chronic diseases which tend to present in the later decades of life, sufferers of headaches span all ages with a large proportion being young, female, and otherwise healthy [1,3]. Headaches are defined as pain in any area of the head and can be categorized into three groups as defined by the International Classification of Headache Disorders (ICHD-III): (1) Primary being a headache without an identifiable cause, including tension, migraine, and chronic cluster; (2) Secondary being a headache with an identifiable cause, including potentially life threatening causes such as vascular disorders or traumatic brain injuries; and (3) Cranial neuropathies [2,4,5]. Cranial neuropathies are defined as pain due to lesions or diseases affecting the cranial nerves, particularly cranial nerves (CN) V, VII, IX, and X, and the upper cervical roots, which transmit pain signals and result in perception of pain in the head and neck. Despite differences in their pathophysiology and presentation, the focus of this review will be on primary type headaches, specifically migraine and chronic cluster headaches (CCH), to reflect the headache sub-types for which neuromodulation has been the most well studied. 

### 1.1. Pharmacotherapy for Migraine 

Migraines encompass a diverse clinical profile, characterized by symptoms that may include headaches, auras, prodromal manifestations, and sensory disturbances. Generally, episodes manifest as a unilateral pulsating headache that persists between 4 and 72 h, exhibiting variation in frequency and intensity [6]. Presently, there is no definitive cure for migraine episodes, rather, therapeutic interventions aim to enhance the patient’s quality of life. From a non-pharmacological perspective, the aim is to recognize and avoid triggers. This goal often necessitates lifestyle adjustments, with emphasis on establishing routines to ensure quality of sleep, consistent exercise, and the prevention of prolonged fasting [7]. Conversely, pharmacological approaches can be categorized into abortive, prophylactic, and specialized treatments overseen by neurologists. Abortive treatments primarily consist of non-steroidal anti-inflammatory drugs (NSAIDs). These have a strong evidence base and function by suppressing cyclooxygenase (COX) isoforms 1 and 2, leading to a reduction in the production of inflammatory agents such as prostaglandins [8]. For moderate to severe migraines, triptans are the primary therapeutic option. These are serotonin agonists targeting the 5-hydroxytryptamine [5-HT] B and D serotonin receptors, suppressing the secretion of vasoactive peptides (VP), substance P (SP), and calcitonin gene-related peptide (CGRP). These compounds have been identified as neuroinflammatory precipitators, stimulating nociceptors affiliated with the trigeminal nerve and transmitting pain signals via the thalamus to the cerebral cortex. Ergot alkaloids are second line and similarly interact with [5-HT] receptors but are non-selective regarding receptor subtypes [9]. 

However, these pharmacological agents are not devoid of limitations. NSAIDs are inadvisable for individuals on anticoagulant therapy, or those with peptic ulcer disease or renal disorders. Given their vasoconstrictive nature, triptans and ergot alkaloids pose risks to patients with coronary artery, peripheral vascular, and cerebrovascular diseases. Furthermore, a critical challenge both clinicians and patients face are ensuring abortive medications are not excessively employed, as this can culminate in medication overuse headaches [8]. The gepants, another emerging class of drugs, antagonize the CGRP receptor directly and have proven efficacy against migraines when compared to placebo; however, further research is needed to evaluate the long-term efficacy and safety profile, as well as comparing the gepants against other established migraine therapies [10,11]. Moreover, monoclonal antibodies targeting CGRP or its receptor have also been developed and shown to be efficacious and well-tolerated for the prevention of episodic and chronic migraine. Given that CGRP is a potent vasodilator, there were concerns about its safety in acute vascular stress such as in stroke or myocardial infarctions [12]. That said, a 2017 randomized, double-blind, placebo-controlled study found no significant adverse cardiovascular effects when using one of the CGRP monoclonal antibodies against the CGRP receptor, erenumab, for patients with stable angina [13] However, the effect in patients where the blood brain barrier is disrupted remains unclear [14]. Additionally, its long half-life and potential to cross the placental barrier remains as a concern for affecting uteroplacental blood flow [15].

Numerous medications have undergone rigorous research for their role in preventing episodic migraines. Importantly, approximately 38% of patients experiencing episodic migraines report positive outcomes from such preventive treatments [16]. One of the first-line treatments for migraine prophylaxis is the beta blocker, of which propranolol predominates given the substantial evidence from research trials highlighting its therapeutic efficacy. Limitations to its use include patients with obstructive lung diseases, atrioventricular conduction defects, and peripheral vascular disease, and it can cause various other behavioral effects as well. Anticonvulsants such as valproic acid and topiramate and tricyclic antidepressants (TCA) such as amitriptyline also play a prominent role in prophylactic migraine therapy [9]. However, their widespread use is limited by the notable risk of adverse reactions associated with this class of drugs. For the anticonvulsants topiramate and divalproex sodium, which are the only two FDA approved anti-epileptic drugs for migraine prevention, careful follow-up testing is required due to risk of pancreatitis, liver failure, teratogenicity, and thrombocytopenia, among various other adverse effects. Additionally, the only TCA that has been demonstrated to have significant evidence affirming its efficacy is amitriptyline, which can be highly sedating and possesses anti-muscarinic and anti-adrenergic properties, among other intolerable effects [17]. In addition, for chronic headaches, OnabotulinumtoxinA (Botox^®^) injections have also proven effective and a randomized double blind trial comparing Botox to topiramate demonstrated superior tolerability [18,19]. The main limitations to its use are cost since it must be administered by a specialist and requires repeat injections every 3 months.

### 1.2. Pharmacotherapy for Chronic Cluster Headache (CCH)

While CCH affects about 0.1% of the population, their clinical course is difficult to predict and troublesome to treat. Unlike other headache subtypes, cluster headaches tend to predominantly affect Caucasian males, and are thought to be mediated by irritation of the trigeminal nerve [20,21]. Cluster headache attacks are described by patients to be excruciating, earning their nickname “suicide headaches”, and tend to be unilateral orbital, retro-orbital, or temporal pain, with cranial autonomic activation causing lacrimation, eye discomfort, nasal congestion, flushing, throat swelling, amongst other symptoms [20,21]. These attacks can include physical agitation or irritation, and last from a few minutes to hours without treatment, though patients may experience several episodic attacks at a time, called bouts [20]. These bouts are cyclical, meaning that patients will experience one or two attacks in a week, followed by nearly daily attacks, before the headaches enter a period of remission [21]. The cyclical nature of these headaches makes them difficult to treat, as medications need to be adjusted to account for changing attack frequency [21]. Therapies include inhalation of 100% oxygen during acute attacks, or the subcutaneous administration of sumatriptan, which has been shown to be the most effective therapy in randomized control trials [21,22]. Sumatriptan is a drug in the triptan class previously described for migraines and remains the first-line treatment for chronic cluster headache [23]. Prophylactic therapies include the calcium-channel blocker verapamil, which is the first-line prophylactic therapy, or the mood-stabilizer lithium, which has a larger side effect profile including potential nephrotoxicity [21]. Some evidence supports the use of other drugs including gabapentin, steroids, and melatonin, though these medications are used less often for CCH [21]. 

### 1.3. Neuromodulation for Headache

Ultimately, though non-pharmacologic and pharmacological treatments exist, headache remains a difficult-to-treat chronic condition for many people. Neuromodulation is an emerging field of biomedical and bioengineering that encompasses implantable and non-implantable technologies, electrical or chemical, for the purpose of improving quality of life and functioning of humans as stated by the international neuromodulation society [24]. Though the applications of neuromodulation are broad, it continues to prove to be a useful and novel approach for managing migraine and chronic cluster headaches, especially for patients who do not respond well to pharmacological interventions or who experience significant side effects from pharmacologic approaches [25]. Research on the use of neuromodulation for the treatment of headache conducted in the last decade has predominantly focused on four neuromodulation techniques, which will be described later in the text: peripheral nerve stimulation (PNS), transcranial magnetic stimulation (TMS), deep brain stimulation (DBS), and spinal cord stimulation (SCS). Though other reviews have focused predominantly on the use of this technology in the context of cluster headaches [25,26], this study elucidates the efficacy of these therapies with special attention to migraine and CHH and provides updates on a rapidly changing field. 

## 2. Materials & Methods

In consultation with an expert research librarian at the University of Wisconsin School of Medicine and Public Health Ebling Library, a search was conducted in PubMed in August of 2023 to survey the current literature on neuromodulation for the treatment of headache. The search included terms related to headaches, neuromodulation, or electro-modulation, and was refined to human research in the English language. In total, the search yielded 1989 results, which were further filtered to include only systematic reviews published between 2022 to 2023 to capture the most up to date and comprehensive research on this topic. Albeit untraditional for narrative reviews to focus on systematic reviews, rather than primary studies, this decision allowed us to summarize the current data more efficiently on a rapidly changing and evolving technology. These review papers were used to guide the following result sub-topics which each focus on a different type of neuromodulation technique, along with any statistically significant data to support their utilization for headache management. The focus of this review is on PNS, TMS, DBS, and SCS as these were the neuromodulation techniques for which there was the most evidence when reviewing systematic reviews published between 2022 and 2023 (Table 1). To enhance our review, the citation lists of review articles were reviewed to find additional research on neuromodulation and supplement the results presented in this paper. Outcome measures reported below include impact on migraine and headache frequency and/or pain intensity, adverse effects of the neuromodulation technique, and associated costs, when available (Table 2).

## 3. Results 

### 3.1. Peripheral Nerve Stimulation (Occipital Nerve Stimulation [ONS]; Vagus Nerve Stimulation [VNS], Trigeminal Nerve Stimulation [TNS])

Peripheral nerve stimulation (PNS) is a broad category of neuromodulation that stimulates various peripheral nerve targets in the face [46]. This therapy was first introduced in the late 1960s and initially involved the surgical placement of a battery but was later modified to a percutaneous approach in the early 2000s [47]. The analgesic benefits of PNS depend on the frequency, pulse width, and intensity modulation; stimulation of the sensory fibers acts to decrease pain perception depending on the placement of the lead in relation to the target area [47].

The most well-studied form of PNS is occipital nerve stimulation (ONS) [48]. ONS is an invasive neuromodulation technique and includes the placement of electrodes near the orbital bones, which are connected to a battery powered pulse generator implanted subcutaneously in the abdomen or in the gluteal region [28]. According to one systematic review of the 45 studies on neuromodulation as preventive treatment for CCH, ONS was the most thoroughly researched [27]. The authors of this review found that when comparing ONS with DBS, ONS had a better safety profile and was overall a better treatment for recurrent CCH [27]. One study included in this review further found that of 65 patients with CCH treated with ONS, there was a significant decrease in mean attack frequency from 17.6 at baseline to 9.5 following treatment [28]. However, although there was a significant reduction in attack frequency using ONS, nearly a quarter of the study participants who underwent ONS experienced adverse outcomes including lead migration or damage, which required remediation, or impaired wound healing, which prolonged recovery [27,28].

Another form of PNS is vagus nerve stimulation (VNS). VNS is a neuromodulation technique that targets the vagus nerve, which is the predominant nerve of the parasympathetic nervous system and helps to regulate physiological homeostasis via autonomic control of vital organs such as the heart [39,48,49]. Initially, VNS was an invasive form of neuromodulation which required the implantation of a device in the cervical spine, resulting in a reduction of its use and thereby leading to the development of newer, less invasive forms [29,39]. The two predominant forms of noninvasive VNS (n-VNS) include cervical vagus nerve stimulation (n-cVNS) and auricular vagus nerve stimulation (n-aVNS) [29]. Of these, n-cNVS was approved by the Food Drug Administration (FDA) for treatment of migraines in 2018, nearly 21 years after the original technology was introduced [29]. This technique simply necessitates the placement of an electrical stimulation device on the concha or tragus of the ear with the goal of stimulating the auricular branches of the vagus nerve, providing analgesic effects [30]. 

One systematic review which looked at six studies on n-VNS for treatment of migraine found that n-VNS did not statistically significantly reduce the number of monthly migraine days for patients [29]. However, when stratified by n–cVNS and n-aVNS, the authors found that n-aVNS did significantly reduce migraine days, suggesting that specific applications of n-VNS may be an effective therapy [29]. Another systematic review which looked at nine studies on n-aVNS similarly found that the therapy helped to reduce the number of migraine attacks [30]. However, the studies included in this review exhibited considerable variability in follow-up periods and study protocols, precluding the evaluation of the data via meta-analysis. Overall, of VNS therapies available, n-aVNS appears to be the most effective in reducing the burden of total migraine attacks/days, however data on the topic is limited to few studies, some with poor data.

Other peripheral nerve stimulation technologies include a non-invasive external trigeminal nerve stimulation (eTNS) technique. This technique involves the placement of a stimulator externally over CN V (i.e., the trigeminal nerve, which provides sensory innervation to the face) for about 30 min daily over a three-month period [26,50]. In one randomized double-blind-sham-controlled study of 67 patients with migraine, eTNS was found to statistically significantly reduce the number of migraine days reported compared to the sham group [26,50]. This therapy was also found to statistically significantly decrease the amount of antimigraine medication used in the intervention group, with no patients reporting adverse effects with this therapy [50]. Another study of 27 patients who failed topiramate treatment for their migraines found a mean decrease in headache days from roughly 9 days per month to 6 days [31]. Less commonly used PNS techniques include sphenopalatine ganglion stimulation and vestibular nerve stimulation, with limited data for their effectiveness described elsewhere [51].

Despite its broad targets and applications, PNS is a relatively safe neuromodulation technique with no side effects appreciated for eTNS. For ONS and VNS, side effects include pain, impaired wound healing, scar formation near the implantation site, and neck stiffness [25,39,48]. Furthermore, there have been reports of repeated surgeries due to lead migration [48]. Unfortunately, the cost for these therapies is not well elucidated in the literature. One study described ONS as a cost-intensive neuromodulation technique with a complicated side-effect profile, though they do not specify the cost [39]. Another study published in 2011 estimated the therapy to cost nearly $30,000, with the generator itself accounting for over half of this cost [49]. Regarding the safety for VNS, one systematic review and meta-analysis revealed no significant difference in occurrence of adverse events between n-aVNS and controls, with the most common side effects being headache, ear pain, and tingling [39]. While the cost effectiveness of this technology for headache has not been clearly elucidated, research on VNS for epilepsy management has shown cost savings for both patients and the healthcare system [52]. 

### 3.2. Transcranial Magnetic Stimulation (TMS)

Transcranial magnetic stimulation (TMS) is a noninvasive neuromodulation technique that alters neural excitability [40]. It involves the placement of a magnetic coil on the scalp. When activated, an electrical current passes through the coil and generates a magnetic field that targets specific areas of the brain, similar to electroconvulsive therapy (ECT) [53]. Importantly, the two therapies differ in that TMS is more focused and can bypass the skull and superficial tissue, requiring milder stimulatory signals [53]. Historically, TMS has been used in the treatment of mood disorders like major depressive disorder and obsessive compulsive disorder, along with its uses for smoking cessation and migraine treatment [40].

Three systematic reviews assessed the utility of repetitive transcranial magnetic stimulation (r-TMS) as a treatment modality for migraine [32,33,34]. One review conducted a random effects analysis of study data from eight separate studies and found that compared to a sham therapy, r-TMS reduced the frequency of migraine attacks, suggesting r-TMS was an effective therapy [32]. Another review found that r-TMS statistically significantly reduced the number of migraine days per month that patients experienced, in addition to a subjective decrease in pain on a pain scale of 0–100 [33]. However, these findings are in direct contradiction to a meta-analysis that found that while r-TMS statistically significantly reduced medication utilization in the studies that they analyzed, the therapy had no effect on pain or total number of migraine days per month [34]. Despite all three studies utilizing the same repetitive stimulation over the left dorsal prefrontal cortex, these findings suggest discrepancies in the literature on the efficacy of r-TMS, as two studies support a reduction in migraine frequency while one meta-analysis found no change. 

One iteration of r-TMS called transcranial direct current stimulation (tDCS) has been posited as a more effective therapy for drug-resistant migraines and CCH as it has been shown to affect larger cortical areas [26]. tDCS is a non-invasive neuromodulation technique that utilizes the placement of two electrodes on the scalp of a patient to transmit weak electrical current thereby modulating cortical excitability [54]. The therapy has polarity-specific effects which can either make neurons hyperpolarized or depolarized which impacts the neuron’s ability to transmit information, including nociception; size, intensity, density, and duration of the stimulation can all also be modified depending on the intended result of the therapy [41,54]. One meta-analysis published in 2023 looked at 31 trials to explore the comparative effectiveness of tDCS against rTMS and nVNS in the prophylactic treatment of migraine [35]. The authors found that when compared to sham intervention alone, tDCS had no statistically significant effect on migraine frequency [35]. However, when compared to rTMS and nVNS, tDCS had a statistically significantly larger effect than either therapy [35]. Between tDCS and rTMS, tDCS was found to have the largest effect on reported pain intensity when compared to sham interventions, but results were not statistically significant when compared to rTMS [35]. These results suggest that tDCS may be favorable for headache intensity and frequency when compared to other neuromodulation modalities or sham therapies, however lack of statistical significance for some of these findings obfuscate the results. 

tDCS is a generally safe and tolerable treatment of chronic migraine. Patients who have undergone the procedure report no short-term side effects, with possible long-term effects including sensitization to therapy or unintended depression of neuronal excitability following prolonged stimulation [41,42]. Cost for this therapy is not well elucidated for migraine, but one study that compared its cost to other motor cortex stimulation techniques such as rTMS found that tDCS was the most cost effective for a single year of treatment [55]. However, these findings were published nearly a decade ago and may be different today.

TMS, as a broader category, has also been shown to be safe. One systematic review and meta-analysis on the safety of TMS for a range of neuropsychiatric disorders, including migraine, found that adverse events with this therapy were rare, though the most reported were headache, followed by facial and scalp pain [40]. There are no current studies to describe the cost effectiveness of this therapy for treatment of headache [56]. 

### 3.3. Deep Brain Stimulation (DBS)

Deep brain stimulation (DBS) is an invasive form of neuromodulation that requires the surgical implantation of a battery-powered electrode into the brain that subsequently delivers electricity at a constant or intermittent pace to various targets [57]. DBS has been shown to be a successful treatment in Parkinson’s and essential tremor when targeting the subthalamic nucleus and globus pallidus [58,59]. Original therapies targeted the ipsilateral posterior hypothalamus for the management of headache. However, subsequent therapies have been described to target other areas in the brain involved in dopaminergic projections that regulate reward, motivation, emotion, and several autonomic processes. These areas include the midbrain ventral and retrorubral tegmentum, sites along the third ventricle wall, and the dorsal longitudinal and mammillotegmental fasciculi [25,60]. Two systematic reviews and one meta-review that assessed the efficacy of DBS for headache looked specifically at its effect on CCH [27,36,37]. The meta-analysis found that in 16 studies totaling 108 cases, there was a statistically significant mean difference in headache attack frequency and intensity following DBS [36]. One systematic review looked at a smaller subset of patients and found that out of the 44 patients studied, there was a 77% mean reduction in headache attack frequency with DBS over a nearly a four-year follow up [37]. Altogether, DBS is a well investigated therapy in the management of headache but given the variety of potential target sites, it has led to a diverse range of experiences and outcomes for patients [27]. 

The safety profile of this therapy for headache management is described in a randomized placebo-controlled double-blind trial of 11 participants, three of which experienced adverse events including infection, transient loss of consciousness, and micturition syncope [43]. More extreme adverse events related to this therapy include one report of subclinical hemorrhage, one report of ipsilateral hemiparesis, and one reported death due to intracranial bleeding [44]. DBS is noted to be the costliest of neuromodulation techniques given the intensive and invasive nature of this neuromodulation technique, with one study estimating that in Europe, the operation would cost a patient nearly 40,000 Euros in 2007 [44].

### 3.4. Spinal Cord Stimulation (SCS)

Spinal cord stimulation (SCS) is an invasive neuromodulation technique that includes the implantation of a stimulation device near the dorsal column, which plays a key role in transmitting information, such as pain signals, to the brain for perception [61]. The SCS device is designed to send out pulsatile information that intercepts and diminishes this signaling [61]. This neuromodulation technique has broad applications and has been shown to be an effective analgesic for patients who suffer from chronic pain when targeting the thoracic and cervical spine [45]. One review article assessed SCS use across four studies on patients with migraine and found that all four studies reported a decrease in migraine intensity and pain [38]. However, given small sample sizes, the studies were limited and did not offer statistical significance for their findings, prompting the authors to conclude that there is low-quality evidence to support the use of SCS for headache [38].

The most cited adverse event associated with this therapy include pain at the site of implantation of the pulse generator [45]. Other adverse events reported include lead displacement and defective devices [45]. Large cost savings associated with this surgery have been described elsewhere for back pain and general analgesic purposes, however no studies to date have explored cost savings related to headache management. 

## 4. Conclusions

Despite the development of numerous pharmacological treatments for headaches, patients still encounter several obstacles. These challenges include but are not limited to variable and inconsistent treatment effectiveness, difficulties in maintaining long-term medication use due to side effects, issues with accessibility and affordability of treatment, and the risk of developing medication overuse headaches. Neuromodulation is an advancing field that holds promise as a supplementary or alternative treatment option for the reduction of headaches and migraine days for chronic sufferers. 

PNS therapies have gained considerable attention for their potential in headache treatment. Its evolution to less invasive techniques has aimed to address patient comfort and accessibility while maintaining its therapeutic efficacy. Of the peripheral nerve stimulation techniques, VNS and TNS are the least invasive while still maintaining strong evidence for their efficacy in reducing headache. Within the VNS techniques, *Song* et al. highlights the importance of targeted stimulation with n-aVNS being superior to n-cVNS. The FDA approving n-cVNS marks a significant milestone, however given the contrasting efficacy between the two different anatomical targets, further refinement in technique and patient stratification is needed. On the other hand, despite ONS demonstrating substantial reductions in headache frequency in the management of CCH, its invasive nature necessitating surgical intervention with possible associated complications of lead migration and hardware damage emphasizes the need for careful patient selection and management. 

TMS is a minimally invasive form of neuromodulation and advances in this technique have resulted in the development of transcranial direct stimulation, allowing for broader targets and more effective therapy in terms of headache management. However, research on the application of TMS and its effectiveness for patients who suffer from headache is limited by small and few studies. Furthermore, evidence for tDCS is promising for migraine and may suggest this modality is superior to rTMS in terms of migraine frequency and pain reduction, however a lack of statistically significant findings in meta-analyses on this topic warrant future research on its effectiveness. The low side effect profile of these non-invasive neuromodulation techniques, along with some evidence for their cost-effectiveness, make them encouraging therapies for headache management, albeit more information is needed. 

DBS, albeit expensive and invasive, has been shown to be an effective option for headache management with the literature providing evidence for significant reductions in the frequency and intensity of headache experienced by patients. That said, this therapy has been associated with severe adverse effects, necessitating stringent risk-benefit analyses for patients prior to recommendation or initiation of this therapy. High costs associated with this neuromodulation technique require further consideration. 

Finally, SCS has been shown to be effective for chronic pain, though evidence is largely inconclusive in terms of its effectiveness in headache or migraine. Reviews of the literature on this neuromodulation technique have concluded that existent evidence is limited by small sample sizes and lack of statistical significance, making it difficult to comment on the utility of this technique for headache. Further investigation with larger patient populations is needed. 

In sum, neuromodulation has developed utility as an alternative treatment for both headaches and migraines. This review has aimed to highlight the status of neuromodulation techniques based on literature published in the last year. Limitations of this review include the limited timespan for which we included papers, our search being restricted to one database, and our decision to focus on review articles to summarize the data, which may introduce bias to our description of the results. Furthermore, we focused on four predominant neuromodulation techniques, but we recognize that there are other invasive and non-invasive neuromodulation modalities that were not covered in this paper due to feasibility of this review and our decision to focus on techniques that were most frequently described in the literature we reviewed. To enhance the literature in the future, a more robust systemic review with meta-analysis is likely needed to summarize and report on the status of the field. Ultimately, as techniques are refined and sample sizes grow, the preferred modalities of this novel technique will continue to offer patients another route to treat via neuromodulation.

## Figures and Tables

**Table 1:** Overview of reviewed sources.

Neuromodulation Technique	Subtype	Acronym	Article Type	Number of Studies or Patients Included	Headache Type Studied	Citation
Peripheral Nerve Stimulation		PNS			CCH ^++^, Migraine	
	Occipital Nerve Stimulation	ONS	Systematic review	45 studies	CCH	Membrilla et al. [27]
			Randomized, double-blind, multicenter, phase 3, electrical dose-controlled trial	65 patients	CCH	Wilbrink et al. [28]
	Vagus Nerve Stimulation (cervical and auricular)	VNS, c-VNS, a-VNS	Systematic review	6 studies	Migraine	Song et al. [29]
			Systematic review	9 studies	Migraine	Fernandez-Hernando et al. [30]
	External Trigeminal Nerve Stimulation	e-TNS	Randomized double-blind-sham-controlled study	67 patients	Migraine	Coppola et al. [26]
			Prospective, multi-center clinical trial study	27 patients	Migraine	Vikelis et al. [31]
Transcranial Magnetic Stimulation		TMS			Migraine	
	Repetitive TMS	r-TMS	Systematic review, random effects analysis	8 studies	Migraine	Zhong et al. [32]
			Systematic review	8 studies	Migraine	Saltychev et al. [33]
			Meta-analysis	5 studies	Migraine	Mohamed Safiai et al. [34]
	Transcranial Direct Current Stimulation	t-DCS	Network meta-analysis	31 studies	Migraine	Chen et al. [35]
Deep Brain Stimulation		DBS			CCH	
			Systematic review and meta-analysis	16 studies	CCH	Murray et al. [36]
			Systematic review	44 patients	CCH	Nowacki et al. [37]
Spinal Cord Stimulation		SCS			Migraine	
			Systematic review	4 studies	Migraine	Finnern et al. [38]

^++^ CCHs stands for chronic cluster headaches.

**Table 2:** Invasiveness of neuromodulation techniques and reported outcomes and adverse events.

Neuromodulation Technique	Subtype	Invasive (I) vs. Non-Invasive (NI)	Cumulative Adverse Events	Primary Outcomes Stratified by Included Papers	Citation
Peripheral Nerve Stimulation	Occipital Nerve Stimulation	NI	Lead migration; impaired wound healing; hardware damage; scar formation at site [27,28].	Improved safety over DBS; better treatment for recurrent CCHs ^++^ compared to DBS.	Membrilla et al. [27]
				Statistically significant decrease in mean attack frequency of CCHs.	Wilbrink et al. [28]
	Vagus Nerve Stimulation (cervical and auricular)	NI	Headache; ear pain; tingling [39].	No statistically significant decrease in monthly migraine days with n-VNS; statistically significant reduction in migraine days with n-aVNS.	Song et al. [29]
				Decrease in number of migraine attacks.	Fernandez-Hernando et al. [30]
	External Trigeminal Nerve Stimulation	NI	None reported [26].	Statistically significant decrease in number of migraine days; statistically significant decrease in antimigraine medication use.	Coppola et al. [26]
				Mean decrease in migraine headache days in patients who failed topiramate therapy.	Vikelis et al. [31]
Transcranial Magnetic Stimulation	Repetitive TMS	NI	Headache; facial pain; scalp pain [40].	Decrease in frequency of migraine attacks.	Zhong et al. [32]
				Statistically significant decrease in number of migraine days per month; subjective decrease in rating of migraines on a pain scale of 0–100.	Saltychev et al. [33]
				Statistically significant decrease in antimigraine medication use; No effect on pain; No effect on total number of migraine days per month.	Mohamed Safiai et al. [34]
	Transcranial Direct Current Stimulation	NI	Sensitization to therapy; depression of neuronal excitability [41,42].	Statistically significant decrease in migraine frequency when compared to rTMS and nVNS; statistically significant decrease in pain intensity when compared to sham.	Chen et al. [35]
Deep Brain Stimulation	–	I	Infection; transient loss of consciousness; micturition syncope; subclinical hemorrhage; ipsilateral hemiparesis; death [43,44].	Statistically significant mean difference in CCH attack frequency and intensity.	Murray et al. [36]
				Mean decrease in CCH attack frequency.	Nowacki et al. [37]
Spinal Cord Stimulation	–	NI	Pain; lead migraine; defective devices [45].	Decrease in migraine intensity and pain.	Finnern et al. [38]
Transcranial Direct Current Stimulation					

^++^ CCHs stands for chronic cluster headaches.
